# Insight into the High-Efficiency Benzo(a)pyrene Degradation Ability of *Pseudomonas benzopyrenica* BaP3 and Its Application in the Complete Bioremediation of Benzo(a)pyrene

**DOI:** 10.3390/ijms242015323

**Published:** 2023-10-18

**Authors:** Xingchen Dong, Siyi Wu, Zihuan Rao, Yaqian Xiao, Yan Long, Zhixiong Xie

**Affiliations:** Hubei Key Laboratory of Cell Homeostasis, College of Life Sciences, Wuhan University, Wuhan 430072, China; andy97@whu.edu.cn (X.D.); 2020202040040@whu.edu.cn (S.W.); rzhmio@whu.edu.cn (Z.R.); 2018102040012@whu.edu.cn (Y.X.); skyjck4@whu.edu.cn (Y.L.)

**Keywords:** *Pseudomonas benzopyrenica* BaP3, PAHs, benzo(a)pyrene, biodegradation, Rhd

## Abstract

Polycyclic aromatic hydrocarbons (PAHs) are common carcinogens. Benzo(a)pyrene is one of the most difficult high-molecular-weight (HMW) PAHs to remove. Biodegradation has become an ideal method to eliminate PAH pollutants from the environment. The existing research is mostly limited to low-molecular-weight PAHs; there is little understanding of HMW PAHs, particularly benzo(a)pyrene. Research into the biodegradation of HMW PAHs contributes to the development of microbial metabolic mechanisms and also provides new systems for environmental treatments. *Pseudomonas benzopyrenica* BaP3 is a highly efficient benzo(a)pyrene-degrading strain that is isolated from soil samples, but its mechanism of degradation remains unknown. In this study, we aimed to clarify the high degradation efficiency mechanism of BaP3. The genes encoding Rhd1 and Rhd2 in strain BaP3 were characterized, and the results revealed that *rhd1* was the critical factor for high degradation efficiency. Molecular docking and enzyme activity determinations confirmed this conclusion. A recombinant strain that could completely mineralize benzo(a)pyrene was also proposed for the first time. We explained the mechanism of the high-efficiency benzo(a)pyrene degradation ability of BaP3 to improve understanding of the degradation mechanism of highly toxic PAHs and to provide new solutions to practical applications via synthetic biology.

## 1. Introduction

Air and soil pollution have become increasingly apparent over the last decades with the development of industrialization and human activities [1]. PAHs are one of the main pollutants in our daily life [2]. They are a group of chemicals with two or more carbon rings. Sixteen types of PAH have been listed as priority pollutants by the Joint FAO/WHO Expert Committee on Food Additives (JECFA) [3]. High-molecular-weight PAHs (HMW PAHs) containing more than four fused aromatic rings have attracted wider attention because of carcinogenesis, teratogenesis, and mutagenesis [4,5]. One of the most carcinogenic PAHs, benzo(a)pyrene, has the highest boiling point and five fused aromatic rings. It is widely used as an exposure label for risk assessments [6,7].

Currently, the main treatment methods for PAH pollutants are physical and chemical oxidation [8,9]. The chemical methods mostly use columns, where a series of oxidation processes are completed [10]. However, the byproducts and intermediate products produced by chemical or physical treatments lead to more serious secondary pollution [11]; these pollutants cannot be completely mineralized (only by approximately 30–40%) [12]. Due to the shortcomings of the physical and chemical oxidation methods, the biodegradation of PAHs is a current research topic and development direction [13,14,15]. The strains used in biodegradation are usually isolated from soil samples that have been seriously polluted by PAHs and other organic substances [16,17,18]. The strains can be practically applied after a series of processes such as domestication and enrichment [19]. Compared with the physical and chemical methods, the biodegradation of PAHs is the most valuable environmental treatment method at present because of its high efficiency, low cost, and mild conditions. There is also no secondary pollution [20,21].

Of the previous reports on the biodegradation of PAHs, there are a small number that focus on HMW PAHs; however, there are few studies on benzo(a)pyrene (the most toxic PAH compound). The benzo(a)pyrene degradation rate of existing strains is not high (approximately 14.8–33.8%) [22,23], and its substrate cannot be completely mineralized. Ring-hydroxylating dioxygenases (RHDs) usually play an important role in the degradation process for the biodegradation of PAHs; they can become a label to search for the strains [24,25,26]. An RHD is usually composed of two subunits. Subunit α is the oxygenase component; it is directly combined with the substrate. Subunit β is the reductase component, and it usually performs an electron transmission function [27]. *Pseudomonas aeruginosa* DN1 is a fluoranthene degradation strain that encodes a group of typical RHDs. It can be used as a research template for the biodegradation of PAHs in the *Pseudomonas* genus [28]. There are few reports on the RHDs that have a high enzyme activity in the degradation of HMW PAHs; the interaction mechanism between RHDs and substrates is also unclear. It is essential to understand the degradation pathways and enzyme activity to successfully apply these strains and their enzymes to environmental protection methods.

*Pseudomonas benzopyrenica* BaP3 is a highly efficient benzo(a)pyrene-degrading strain that was previously isolated by our group [29]. Compared with existing strains [12,22,23,30], strain BaP3 has a higher degradation rate and capacity; it can degrade benzo(a)pyrene by approximately 50% within 6 days. All these advantages render strain BaP3 a suitable research tool to understand the biodegradation characteristics of HMW PAHs. In this study, we used *Pseudomonas benzopyrenica* BaP3 due to its high-efficiency benzo(a)pyrene degradation ability, and we successfully clarified its extraordinary HMW PAH degradation mechanism. We identified that the RHDs of strain BaP3, particularly the gene *rhd1*, were involved in the process of benzo(a)pyrene degradation via bioinformatics, molecular biology, genetics, and biochemistry methods. A novel RHD, named Rhd1, which could interact with benzo(a)pyrene, was purified, and the enzyme activities were determined. The enzyme had a hydrophobic cavity and a higher benzo(a)pyrene degradation activity than the existing enzymes. Based on the principles of synthetic biology, *rhd1* was expressed in *Pseudomonas donghuensis* HYS, a strain exhibiting high yields of siderophores [31]. For the first time, we successfully and completely mineralized benzo(a)pyrene. We created a new enzyme with a high degradation capacity for benzo(a)pyrene that enabled the complete biodegradation of HMW PAHs. This may enhance the effectiveness of their environmental governance.

## 2. Results

### 2.1. Annotation of Two RHDs above the Genome Sequence of Strain BaP3

**Figure 1:**
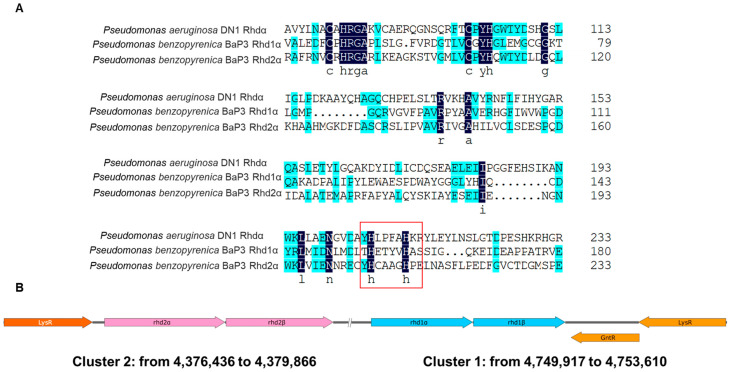
The multiple alignments of Rhd subunit alpha (**A**) and two clusters containing the genes *rhd1* and *rhd2* (**B**). Compared with the reported Rhd in *Pseudomonas aeruginosa* DN1, three proteins had similar active center residues The conserved amino acid residues in all of the sequences are indicated with black and blue backgrounds. The residues of the catalytic active center confirmed in *P. aeruginosa* DN1 are marked in the red box.

The whole genomic map of *Pseudomonas benzopyrenica* BaP3 is shown in Figure S1. The sequence can be obtained from the NCBI database under accession number JAPFGF000000000. Based on the completed genome, the active center sequence of Rhdα from *P. aeruginosa* DN1 was selected for multiple alignments. According to the protein sequence of the multiple alignments (Figure 1A), Rhd1α and Rhd2α contained typical key residue sequences of RHD that could have been related to the degradation of benzo(a)pyrene. The alpha subunits of the two RHDs were adjacent to the beta subunits, and the arrangements were typical RHD clusters (Figure 1B).

### 2.2. RHDs Are the Main Genes Involved in the Degradation of Benzo(a)pyrene in Strain BaP3

**Figure 2:**
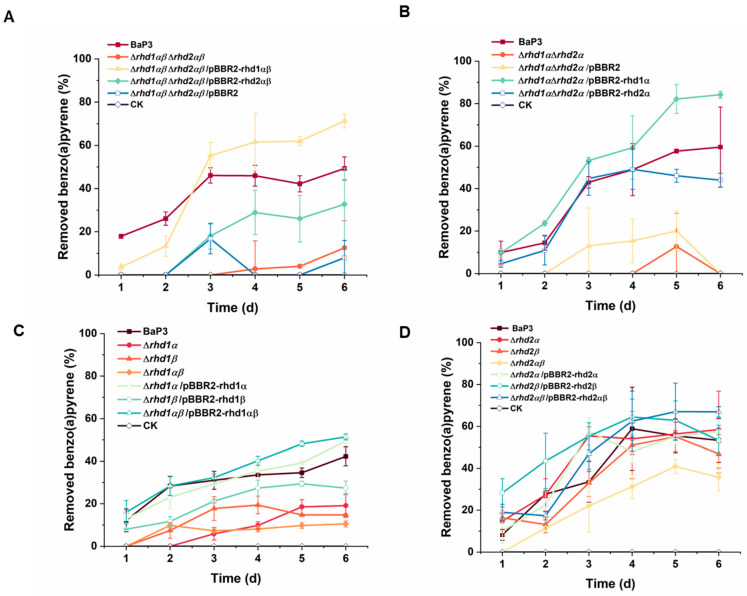
Degradation capacity of benzo(a)pyrene (**A**–**D**). The degradation efficiency of the wild-type, deletion, and complement strains was tested in MSM containing 15 mg/L benzo(a)pyrene at 30 °C and 200 rpm (10 μg/mL kanamycin was added when plasmids were used). The values proposed were the average ± standard deviation of three independent experiments (*n* = 3). CK represents the cultures without bacteria.

To determine the roles of genes *rhd1αβ* and *rhd2αβ*, a mutant of two RHDs (Δ*rhd1αβ*Δ*rhd2αβ*) was constructed (Figure S2A). When *rhd1αβ* and *rhd2αβ* were deleted, the mutant strain almost completely lost the ability to degrade benzo(a)pyrene (Figure 2A). When *rhd1αβ* was complemented into strain Δ*rhd1αβ*Δ*rhd2αβ*, the degradation ability was recovered. The complement of *rhd2αβ* could not regain the high degradation efficiency of the strain.

To verify the functions of the alpha subunits in the two RHDs, the alpha subunits were deleted at the same time (Figure S2B). Similarly, strain Δ*rhd1α*Δ*rhd2α* lost the ability to use benzo(a)pyrene (Figure 2B). When *rhd1α* was complemented into strain Δ*rhd1α*Δ*rhd2α*, the ability was recovered, and the efficiency increased to approximately 80%. The efficiency of the complement of *rhd2α* was about 40%.

Single-knockout mutants of alpha or beta subunits (Δ*rhd1α*, Δ*rhd1β*, Δ*rhd2α*, and Δ*rhd2β*) and double-knockout mutants (Δ*rhd1αβ* or Δ*rhd2αβ*) were also obtained (Figure S2C–H). Compared with the wild type, the degradation of mutants Δ*rhd1αβ*, Δ*rhd1α*, and Δ*rhd1β* was significantly reduced (Figure 2C). The degradation of strain Δ*rhd1αβ* reduced to approximately 10%, almost completely losing its ability. Its ability could be recovered after complementing. The efficiencies of strain Δ*rhd1α*/pBBR2-rhd1α and Δ*rhd1αβ*/pBBR2-rhd1αβ were higher than the wild type. Based on the growth curves, the mutants and wild type demonstrated a similar growth tendency in an LB medium. We concluded that the reduction in degradation ability was caused by a gene deletion (Figure S3). For *rhd2*, the deletion of each subunit or both subunits did not significantly influence the degradation ability (Figure 2D).

### 2.3. Rhd1α Is Suitable for the Degradation of Benzo(a)pyrene

**Figure 3:**
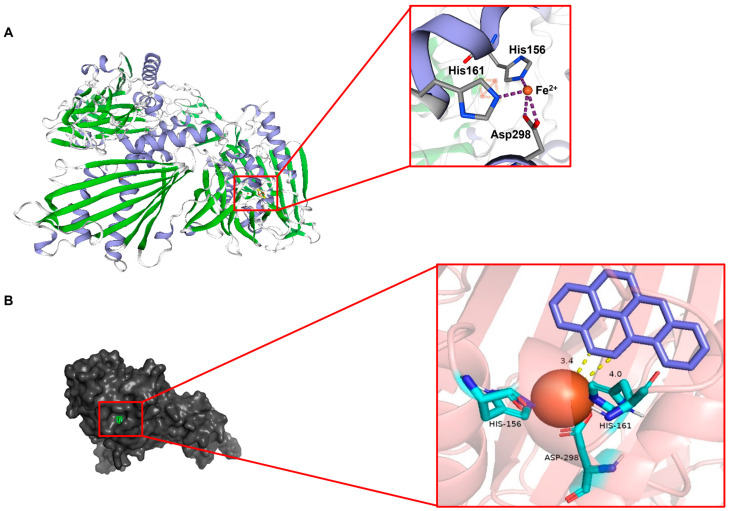
Homology model of Rhd1α and predicted active site of Rhd1α (**A**), molecular docking of Rhd1α and benzo(a)pyrene and pattern of C_11_-C_12_ carbon interacting with Rhd1α (**B**). The model was constructed using the SWISS-MODEL databased based on the published structure of 3gl2.1.A. The α-helixes are colored purple, and the β-sheets are colored green. It had a typical predicted active site, formed with H156, H161, D298, and Fe (II). The docking results revealed that benzo(a)pyrene (colored green in (**B**)) was located in the cavity of the active site. C_11_ and C_12_ carbons were the closest atoms.

Based on the published crystal structure 3gl2.1.A, homology modeling of Rhd1α was conducted using the SWISS-MODEL database (Figure 3A). The template protein was a Rieske oxygenase that was similar to Rhd1α (Figure S4). After modeling, a Ramachandran plot was used to test the distribution rationality of the amino acid residues in this structure (Figure S5A). The Z-score values of the Rhd1α was distributed within the graph range plotted by the Z-score values of the known proteins (Figure S5B). The resulting structure of Rhd1α was a homo-trimer and contained a predicted active site that was composed of two histidine residues (His156 and His161) and one aspartic residue (Asp 298) (Figure 3A).

An analysis of the molecular docking was conducted based on the AutoDock software version 4. Benzo(a)pyrene was used as a substrate ligand (produced by the ChemDRAW software version 19.0), and Rhd1α was used as the macromolecular receptor. After 100 docking programs based on the most likely site and the lowest energy, the best binding result was obtained (Figure 3B). The binding site of Rhd1α and the substrate were located in the cavity constructed by His156, His161, Asp298, and Fe (II). The C_11_ and C_12_ carbon atoms on the benzo(a)pyrene ring were the closest to Fe (II); the distances were 3.4 Å and 4.0 Å, respectively (Figure 3B). The binding energy of this complex structure was −7.66 kcal/mol.

### 2.4. Residues H156, H161, and D298 Are Essential for Rhd1α

**Figure 4:**
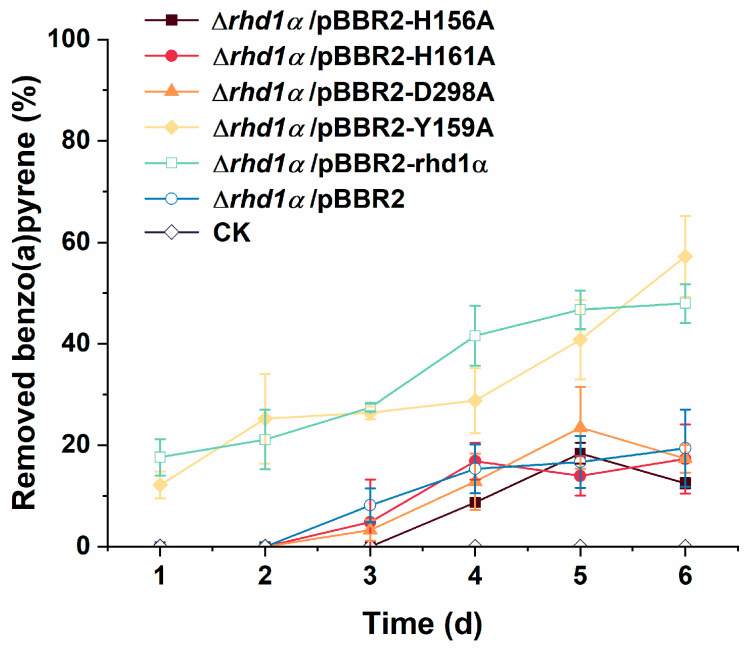
The degradation curves of point substitutions and other control groups. The degradation efficiency was tested in MSM containing 15 mg/L benzo(a)pyrene at 30 °C and 200 rpm (10 μg/mL kanamycin was added when plasmids were used). The values proposed were the average ± standard deviation of three independent experiments (*n* = 3). CK represents the cultures without bacteria. The substitutions that could not form an active site lost the degradation ability of benzo(a)pyrene and efficiency was close to that of strain Δ*rhd1α*/pBBR2. The substitution of Y159 still had capacity and its efficiency was close to that of Δ*rhd1α*/pBBR2-rhd1α.

The active site of the Rhd1α interacting with the substrate is indispensable for elucidating the catalytic mechanism of enzyme interaction with substrates with high accuracy. To confirm that the residues that were predicted by molecular docking played crucial roles during the degradation of benzo(a)pyrene, point substitutions of H156A, H161A, and D298A were produced. First, homology modeling of these point-substitution proteins was performed. When the predicted active residues were substituted by alanine, the active site could not be formed (Figure S6A–C). The Y159 residue was located near the active center, but it has not been reported to participate in the formation of the active center. We chose it as a control group. When Y159 was substituted by alanine, the predicted active site could still be constructed (Figure S6D). In addition to the bioinformatics analysis, these residues were substituted by alanine via overlap PCR. Compared with the removal percent of the wild type, a similar degradation trend was observed in mutants H156A, H161A, D298A, and Δ*rhd1α*/pBBR2 (Figure 4). Mutant Y159A maintained its degradation ability. It was confirmed that the residues predicted by molecular docking simulation played decisive roles during catalysis. On the basis of these results, Rhd1α was used in the degradation process of benzo(a)pyrene in strain BaP3. The active site of Rhd1α was assembled by H156, H161, D298, and Fe (II).

### 2.5. The In Vitro Active Detection of Rhd1

The genes *rhd1α* and *rhd1β* were amplified and expressed in pGEX-4T-1 in *E. coli* BL21 (DE3). The expected molecular masses of GST-Rhd1α and GST-Rhd1β were 65.8 kDa and 61.4 kDa. The heterologous expression and purification of GST-Rhd1α and GST-Rhd1β were successful, with molecular masses of 65 kDa and 60 kDa, respectively (Figure S7A,B).

The purified Rhd1α protein had characteristic absorption peaks at 323 nm, 420 nm, and 454 nm (Figure S7C). According to previous research, these absorption peaks are typical characteristics of the oxygenase components of RHD [27]. Rhd1β had absorption peaks at 275 nm, 345 nm, 423 nm, 460 nm, 485 nm (shoulder), and 548 nm (shoulder) which were typical peaks for the reductase components of RHD (Figure S7D) [27].

**Figure 5:**
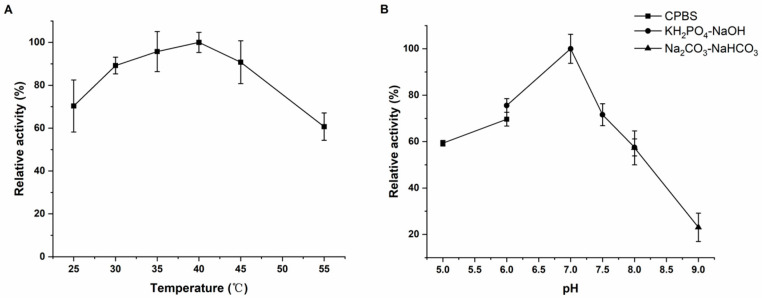
The enzyme activity was tested at different temperatures (**A**) and pH values (**B**). The enzyme activity curves were indirectly reflected by the consumption of NADH. The values proposed were the average ± standard deviation of three independent experiments (*n* = 3). The results revealed that the Rhd1 had the highest enzyme activity at 40 °C and pH value of 7.0.

Purified recombinant proteins GST-Rhd1α and GST-Rhd1β were detected as being positive for benzo(a)pyrene degradation enzyme activity. To determine the activity of the purified proteins in vitro, an WST-8 kit was used to indirectly reflect the activity of RHD by measuring the consumption of NADH. The enzyme activities of the purified proteins were determined at different temperatures and pH values. Rhd1α and Rhd1β had the highest enzyme activity at 40℃ and a pH value of 7.0 (Figure 5A,B).

### 2.6. The Accumulation of Phenylacetic Acid May Be the Reason Why Benzo(a)pyrene Could Not Be Completely Degraded by Strain BaP3

**Figure 6:**
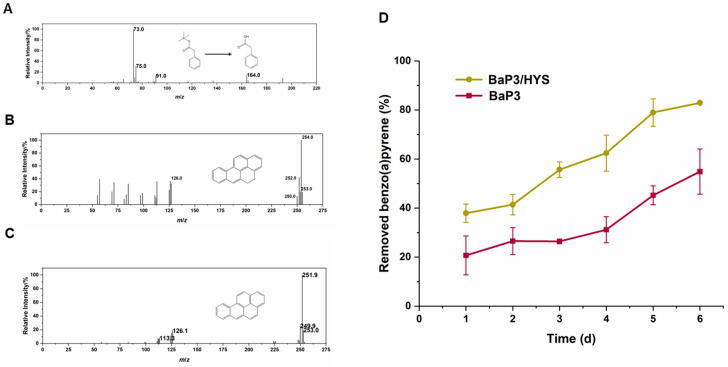
The mass spectra of benzo(a)pyrene degradation process (**A**–**C**) The co-metabolism of strain BaP3 and strain HYS (**D**). The degradation efficiency was tested in MSM containing 15 mg/L benzo(a)pyrene at 30 °C and 200 rpm. The values proposed were the average ± standard deviation of three independent experiments (*n* = 3). The relative intensity of ion peaks was shown in each mass spectrum. As phenylacetic acid accumulated, the gene *paaK* was not explored in the genome sequence of BaP3. This could be the reason why strain BaP3 could not completely mineralize the substrate. The hypothesis was confirmed by the co-metabolism of BaP3 and HYS.

The wild-type and the complement strains could not completely mineralize the substrate (Figure 2). The degradation efficiency usually ceased at approximately 40–60% in a week and would not increase. To investigate the accumulation of intermediate products, the toxicity, or the feedback inhibition of the degradation process, GC-MS was used to determine the intermediate products during the benzo(a)pyrene degradation process. BSTFA: TMCS (99:1) was used to derive the samples before the examination. Three obvious substances were detected via GC-MS in the wild-type strain. These were benzo(a)pyrene, dihydrobenzo(a)pyrene, and phenylacetic acid (Figure 6A–C). Dihydrobenzo(a)pyrene and phenylacetic acid could not be detected in strain Δ*rhd1αβ*Δ*rhd2αβ*. This result supported our hypothesis that RHDs were the main genes involved in the degradation of benzo(a)pyrene in strain BaP3.

To confirm whether the accumulation of phenylacetic acid inhibited the degradation of benzo(a)pyrene, strain BaP3 was co-cultured with *P. donghuensis* HYS (a strain that can use phenylacetic acid) to detect the removal rate of benzo(a)pyrene (Figure 6D). Compared with strain BaP3 cultured alone, the degradation efficiency of the co-cultured group increased from approximately 50% to approximately 80% in 6 days. The accumulation of phenylacetic acid may have been the reason why benzo(a)pyrene could not be completely degraded.

### 2.7. Synthetic Biology Is a Beneficial Tool to Increase the Degradation Efficiency of PAHs

**Figure 7:**
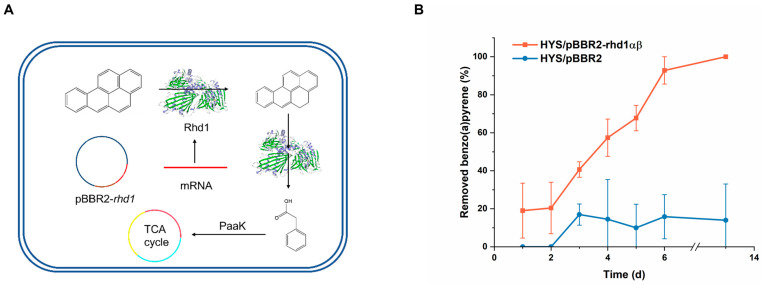
The coupled pathway of degradation (**A**). The degradation efficiency of recombination strain HYS/pBBR2-rhd1 (**B**). Degradation efficiency was tested in MSM containing 15 mg/L benzo(a)pyrene at 30 °C and 200 rpm (10 μg/mL kanamycin was added when plasmids were used). The values proposed were the average ± standard deviation of three independent experiments (*n* = 3). HYS/pBBR2 was set as the control group. The recombination strain had the highest efficiency and could completely mineralize benzo(a)pyrene, whereas the degradation ability of HYS/pBBR2 maintained levels of 0 to 10% in two weeks.

Based on the above results, we identified that the accumulation of phenylacetic acid in strain BaP3 caused the interruption of degradation. We designed a coupling for the metabolic pathways according to the principles of synthetic biology (Figure 7A). Once the intermediate product was consumed, the pathway was completed, and benzo(a)pyrene could be completely removed.

To couple the benzo(a)pyrene ring-opening ability of BaP3 with the phenylacetic acid metabolic ability of HYS, strain HYS was transformed with pBBR2-rhd1 and cultured in MSM (with benzo(a)pyrene and kanamycin added). As strain HYS containing an empty plasmid was set as the control group (Figure 7B). Strain HYS/pBBR2-rhd1 degraded more than 90% of the benzo(a)pyrene in 6 days. After incubation for two weeks, the concentration of benzo(a)pyrene in the MSM was lower than the detection limit; therefore, we considered benzo(a)pyrene to be completely mineralized. Through the rational coupling of the metabolic pathways, we achieved the goal of completely degrading the benzo(a)pyrene of HMW PAHs for the first time.

## 3. Discussion

*Pseudomonas benzopyrenica* BaP3 degraded benzo(a)pyrene with high efficiency; there were two RHDs in strain BaP3. As RHDs play important roles in other PAH-degrading strains [32], whether these two RHDs participated in the benzo(a)pyrene degradation process or not and understanding main functions are valuable topics of research. Rhd1αβ and Rhd2αβ co-functioned in the removal of benzo(a)pyrene; Rhd1αβ played a major role. Alpha subunits are the key component of RHDs. The mutant that was deleted from the alpha subunit had a lower degradation capacity than the mutant of the beta subunit. The results were consistent with previous studies that suggested that the alpha subunit plays a key role in RHDs [28].

The structure of Rhd1α shared a 37.46% similarity with the template protein. Previous studies have confirmed that homologous proteins have a similar backbone structure to templates when they share a similar amino acid sequence greater than 30% [33]. Different enzymes have different active sites; the majority of RHDs have a conservative active site constructed with His-His-Asp and Fe (II) [34]. The active site of Rhd1α was confirmed via molecular docking and point substitution.

The result of molecular docking matched the distance range for many enzyme–substrate interactions. The binding energy was consistent with previous reports that suggested the possibility of enzyme–substrate interactions [35,36]. Compared with the template protein, many residues around the active site changed in Rhd1α (Figure S4). We concluded that these changes to the binding site resulted in it becoming hydrophobic and the cavity becoming nonpolar. PAHs—especially benzo(a)pyrene—have high boiling points, high melting points, low vapor pressure, and low water solubility [37]. The composition of the active center influenced the enzyme’s catalytic rate [38]. As strain BaP3 could degrade a five-ring PAH benzo(a)pyrene, the difference between the amino acid residues of the active site among the RHDs explained the degradation efficiency and substrate discrimination to an extent [39]. The nonpolar cavity of Rhd1α was beneficial for the binding substrate.

Dihydrobenzo(a)pyrene and phenylacetic acid were detected via GC-MS; they were located in the initial position and rear position of the network, respectively [40]. The generation of dihydrobenzo(a)pyrene was the first step reaction of the whole network and was catalyzed by RHDs; the substrates of RHDs are not usually specific [32]. The metabolism of phenylacetic acid usually begins with the gene *paaK* [41]. However, the local blast comparison of strain BaP3 failed to discover any relevant genes. We inferred that the reaction process from benzo(a)pyrene to phenylacetic acid may have been catalyzed by RHDs. The absence of a metabolic pathway for phenylacetic acid in the BaP3 genome sequence caused the accumulation of phenylacetic acid, which in turn prevented the strain from completely degrading benzo(a)pyrene. *P. donghuensis* HYS [31], which can use phenylacetic acid to synthesize 7-hydroxytropolone (a type of siderophore), has been extensively studied by our research team; the relationship between synthesis and degradation is also understood [42]. The co-culture of strain BaP3 and HYS proved the above conjecture.

Synthetic biology originated at the turn of the millennium; the theory facilitates cell and molecular biology advances to productive ends [43]. In recent years, synthetic biology has been a tool for novel secondary metabolites [44], targeted protein degradation [45], plant metabolism [46], etc. As an effective environmental microbial resource, *Pseudomonas donghuensis* HYS has good environmental adaptability [47] and exhibits high yields of siderophores with Fe (II) [31], which is an important component of the RHD active center. It is useful to create a strain from *P. donghuensis* HYS that can not only adapt to the environment, but also control PAH pollution. Based on the synthetic biology theory, a recombinant strain was proposed. This strain could completely mineralize benzo(a)pyrene.

In conclusion, we revealed a novel RHD produced by *Pseudomonas benzopyrenica* BaP3. We proved that the *rhd* gene had a crucial position in the degradation progress of strain BaP3. RHDs catalyze the initial oxidation reaction step by transforming the oxygen atoms of O_2_ to PAH substrates [48]. We proposed an interaction model with a hydrophobic cavity; molecular docking revealed that it could tightly bind to benzo(a)pyrene (a five-ring PAH). The model was confirmed via point substitutions. The enzyme activity determination demonstrated that the purified recombinant protein Rhd1 reflected the significant catalytic degradation ability of benzo(a)pyrene. We also improved the utilization capacity of benzo(a)pyrene. The accumulation of an intermediate product was the reason why strain BaP3 could not completely degrade benzo(a)pyrene; this was supported by GC-MS and a co-culture. When *rhd1* was transformed into *P. donghuensis* HYS (a strain that can degrade phenylacetic acid), benzo(a)pyrene could be fully consumed. As benzo(a)pyrene is considered to be one of the most carcinogenic PAHs, our study enhances the understanding of the microbial degradation of HMW PAHs not only in molecular mechanisms but also in potential practical applications.

## 4. Materials and Methods

### 4.1. Strains, Plasmids, and Culture Media

The bacterial strains and plasmids used in this work are listed in Tables S1 and S2. *Pseudomonas benzopyrenica* BaP3 was isolated by our group from soil samples collected from Huangshi, Hubei Province, China, in July 2019. Strain BaP3 can be obtained from the China Center for Type Culture Collection (CCTCC) and Japan Collection of Microorganisms (JCM) under accession numbers CCTCC AB 2022379 and JCM 35914. *P. donghuensis* HYS, a strain that can degrade phenylacetic acid, was preserved in our laboratory. Strain HYS can be obtained from CCTCC under accession number CCTCC AB 2012141. *Escherichia coli* DH5α, S17-1 (λpir) [49], and BL21 (DE3) were used for the plasmid construction, gene deletion, and protein expression, respectively. Luria–Bertani (LB) (per liter of distilled water, comprising 5 g NaCl, 5 g yeast extraction, and 10 g peptone), a mineral salt medium (MSM) modified with the reagents mentioned by Palma et al. [50], and benzo(a)pyrene were dissolved in acetone and added to the MSM to a final concentration of 15 mg/L. This mixture was used to culture the bacteria. Antibiotics were added to the culture medium based on the resistance genes carried by plasmids or strains at the following concentration: 50 μg/mL kanamycin, 10 μg/mL gentamicin, 10 μg/mL chloramphenicol, and 100 μg/mL ampicillin.

### 4.2. Whole-Genome Analysis

The genome sequence of strain BaP3 was obtained from the Illumina Nova Seq platform at Suzhou PANOMIX Biomedical Tech Co., Ltd. (Suzhou, China) and assembled via SPAdes [51]. This whole-genome shotgun project was deposited at DDBJ/ENA/GenBank under the accession number JAPFGF000000000. The version described in this paper is version JAPFGF010000000. The NR database was used to annotate the protein-coding genes. The RHDs were aligned using BLASTP [52].

### 4.3. Gene Deletion and Complement

The primers listed in Table S3 were used to amplify the upstream and downstream fragments via overlapping PCR. The suicide vector pEX18Gm [53] was used to construct the knockout strains based on a homologous recombination, as described by a previous report [54]. The correct strains were confirmed via PCR and sequencing. For the complement, the Shine–Dalgarno sequences and open reading frames (ORFs) of the target genes were amplified and combined with pBBR1MCS-2 [55]. The recombinant vector was transformed into the target strain via electroporation.

### 4.4. Determination of Degradability and Growth Curve

All the strains were cultured in the LB medium for about 14 h. The *OD_600_* of the bacterial solution was determined and adjusted to about 1.0. To determine the degradability, 1 mL bacterial solution was inoculated in the MSM supplemented with 15 mg/L benzo(a)pyrene (96%; purchased from RHAWN, China) as the sole carbon source at 30 °C and 200 rpm for 6 days. The degradation efficiency was estimated at a wavelength at 295.5 nm using an ultraviolet spectrophotometer UV-2550 (SHIMADZU, Japan). High-performance liquid chromatography (Agilent HPLC 1200, Santa Clara, CA, USA) was used to determine the concentration. To determine the growth curves, all strains were cultured in the LB medium at 30 °C at 200 rpm, and the *OD_600_* was measured every 2 h. All experiments were repeated three times, and the average results were presented with standard errors.

### 4.5. Homology Modeling and Molecular Docking of Rhd1α

To analyze the structure of Rhd1α, a published crystal structure of 3gl2.1.A (https://swissmodel.expasy.org//templates/3gl2.1, accessed on 26 April 2022) was selected as the template for homology modeling by using the automatic search function in SWISS-MODEL. The amino acid sequence of Rhd1α and the published crystal structure 3gl2.1.A were analyzed, and then the model was built using the automatic program in SWISS-MODEL. The model was examined using a psi/phi Ramachandran plot [56] and ProSA-web [57]. To elucidate the binding mechanism and binding site of Rhd1α and benzo(a)pyrene, molecular docking was completed using the AutoDock software version 4 [58]. The structure of the benzo(a)pyrene was produced by the ChemDRAW software version 19.0.

### 4.6. Site-Directed Mutation

The site-directed mutations were achieved through overlapping PCR with the primers shown in Table S3. Plasmid pBBR1MCS-2 was used. The recombinant vectors were transformed into strain Δ*rhd1α* via electroporation.

### 4.7. Purification of Rhd1α and Rhd1β Protein

Rhd1α and Rhd1β were expressed and purified with plasmid pGEX-4T-1 in *E. coli* strain BL21 (DE3). Strain BL21-rhd1α was cultured overnight in 5 mL of the LB medium containing 100 μg/mL ampicillin at 37 °C and 200 rpm. Next, 500 μL of a bacterial solution was inoculated into 50 mL LB containing 100 μg/mL ampicillin in a 250 mL flask. Five flasks were used, and 250 mL of solution was obtained. After shaking at 37 °C and 200 rpm for approximately 1.5 h, the *OD_600_* reached approximately 0.6. Then, 0.1 mmol/L (final concentration) IPTG was added into the flasks, which were shaken at 20 °C and 140 rpm for 10 h. The cells were harvested via centrifugation (at 8000× *g* and 4 °C for 15 min), and the cells were resuspended with 5 mL Tris/NaCl buffer (0.5 mol/L NaCl, 0.05 mol/L Tris, 0.7 mol/L glycerol, and the pH value was adjusted to 8.0). The resuspended cell solution was broken via ultrasonic cell crushing apparatus at 140 w until the solution became transparent. The solution was centrifuged at 8000× *g* and 4 °C for 30 min, and the supernatant was collected. After binding with 3 mL glutathione agarose, the Tris/NaCl buffer was used to remove miscellaneous proteins, and 5 mmol/L and 10 mmol/L of a GSH–Tris/NaCl buffer were used to obtain the purified proteins, respectively. Each of the eluting components was detected via SDS-PAGE. The recombinant protein was purified and concentrated via ultrafiltration.

### 4.8. Characteristic Absorption Peaks and Enzyme Activity

To determine the characteristic absorption peak of the purified proteins Rhd1α and Rhd1β, a UV-2550 (SHIMADZU, Japan) was used for full-band scanning. For the enzyme activity, the enzyme reaction system contained 0.5 mmol/L benzo(a)pyrene, 0.1 μmol/L Rhd1α, 0.1 μmol/L Rhd1β, 0.5 μmol/L FMN, 0.1 mmol/L Fe (NH_4_)_2_·(SO_4_)_2_·6H_2_O, and 10 mmol/L NADH [27]. For the determination of the optimum temperature, reaction systems were heated using a water bath at 25, 30, 35, 40, 45, 50, and 55 °C, respectively. For the pH values, the reaction solutions were pH 5.0–6.0 citric acid-Na_2_HPO_4_ (CPBS), pH 6.0–8.0 KH_2_PO_4_-NaOH, and pH 8.0–9.0 Na_2_CO_3_-NaHCO_3_. The reaction lasted for 5 min, and the concentration of NADH was determined using a WST-8 kit (Beyotime Biotechnology, China). The activity of Rhd1 was determined by measuring the consumption of NADH. The condition with the highest enzyme activity was used as a reference point (100%) to calculate the relative enzyme activity under each experimental condition.

### 4.9. GC-MS Analysis

After being cultured for 7 days, 10 mL ethyl acetate was used to extract the sample. This process was repeated one more time, and 20 mL of the solution was obtained. This was dried using anhydrous sodium sulfate. The extraction liquid was then concentrated to approximately 1 mL by using a rotary evaporator. The liquid was transferred to a threaded Teflon tube and blown to nearly dry a under nitrogen flow. To redissolve the product, 100 μL hexane was added. Then, 150 μL BSTFA: TMCS (99:1) and a water bath at 60 °C for 1 h were used to derivatize the sample. The solution was filtered using a 0.22 μm nylon membrane for the determination. The test was performed under the following conditions. A total of 1 μL of the filtered sample was injected into GC-MS equipment (Thermo TSQ 8000 Evo, Waltham, MA, USA) with helium as the carrier gas at a constant flow of 1 mL/min. The inlet temperature was set at 300 °C. The oven temperature was held at 70 °C for 2 min; this was gradually increased to 320 °C at 10 °C/min and then held for 5 min. The mass spectrometer was set with an ionization energy of 70 eV and a transfer line temperature of 300 °C was used. Metabolites were identified from the GC-MS database using Thermo Xcalibur software version 4.1 [59].

### 4.10. Co-Metabolism and Heterologous Expression

Co-metabolism and heterologous expression were attempted to verify the benzo(a)pyrene degradation ability of *rhd1* and improve the degradation capacity based on the principles of synthetic biology. Strain HYS had phenylacetic acid metabolism-related genes; these genes could not be found in strain BaP3. Briefly, strain BaP3 and strain HYS were cultured together in MSM supplemented with 15 mg/L benzo(a)pyrene as the sole carbon source for 6 days. the degradation efficiency was monitored, as described above. For the heterologous expression, the rhd1-F and rhd1-R primers were used to amplify the fragment of *rhd1* (including the SD sequence) ligated with plasmid pBBR1MCS-2. The recombinant vector pBBR2-rhd1 was transformed into strain HYS via electroporation. The degradation efficiency was tested, as described above. A strain of HYS with an empty plasmid was used for the control group.

## Data Availability

All data generated or analyzed during this study are included in this published article (and its Supplementary Information files).

## Supplementary Materials

The supporting information can be downloaded at: https://www.mdpi.com/article/10.3390/ijms242015323/s1, references [49,53,55].
