# Supplementary material for: Insight into the High-Efficiency Benzo(a)pyrene Degradation Ability of Pseudomonas benzopyrenica BaP3 and Its Application in the Complete Bioremediation of Benzo(a)pyrene
Source: Int J Mol Sci. 2023 Oct 18;24(20):15323. doi: 10.3390/ijms242015323 (PMC10607497; doi:10.3390/ijms242015323)
Supplement: Supplementary file 1 [file ijms-24-15323-s001.zip › ijms-2648524-supplementary.pdf]

Supplementary Materials

Insight into the High-Efficiency Benzo(a)pyrene Degradation Ability of *Pseudomonas benzopyrenica* BaP3 and Its Application in the Complete Bioremediation of Benzo(a)pyrene

Xingchen Dong, Siyi Wu, Zihuan Rao, Yaqian Xiao, Yan Long and Zhixiong Xie *

Hubei Key Laboratory of Cell Homeostasis, College of Life Sciences, Wuhan University, Wuhan 430072, China; andy97@whu.edu.cn (X.D.); 2020202040040@whu.edu.cn (S.W.); rzhmio@whu.edu.cn (Z.R.); 2018102040012@whu.edu.cn (Y.X.); skyjck4@whu.edu.cn (Y.L.)

* Correspondence: zxxie@whu.edu.cn

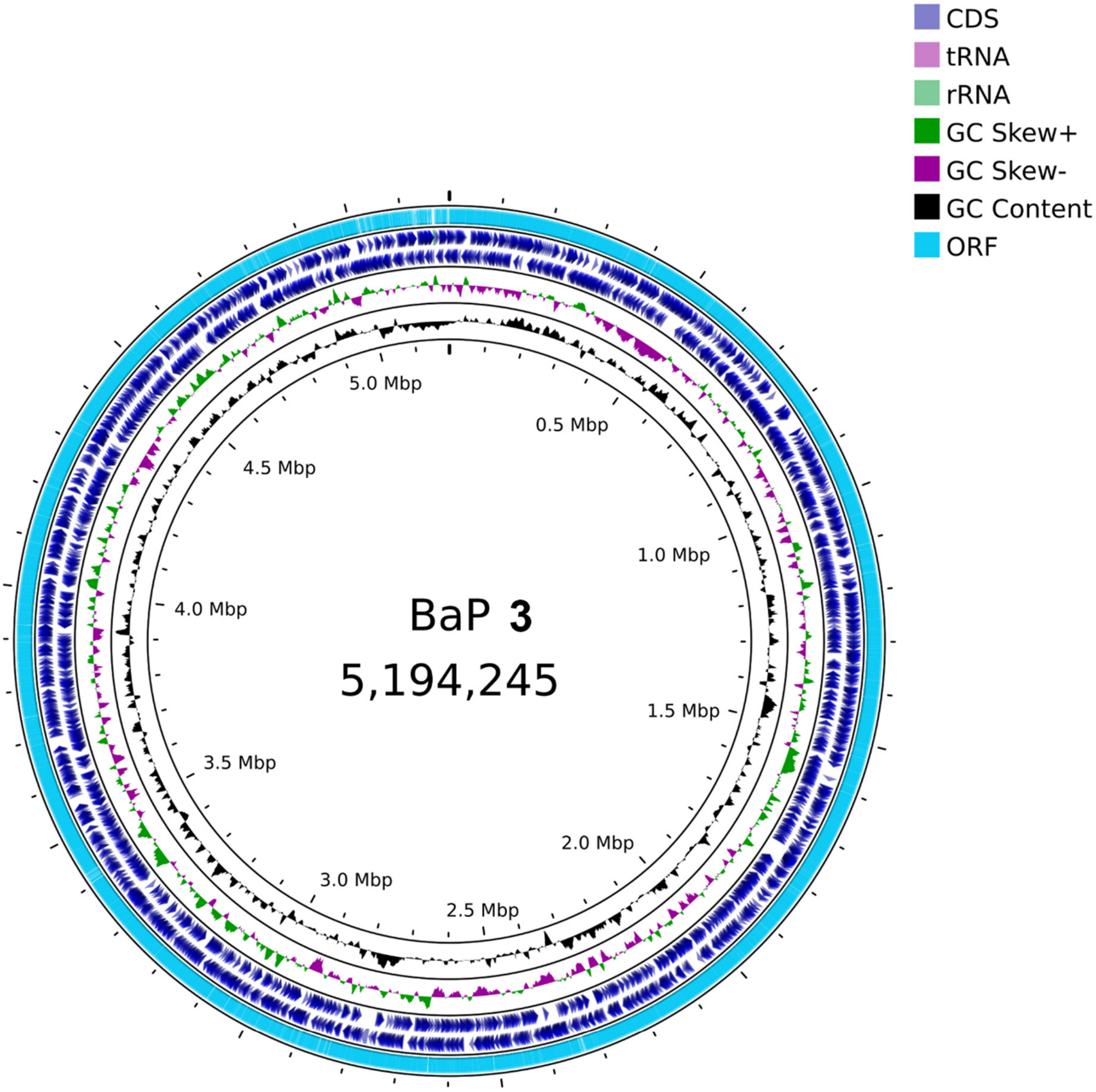

Figure S1. The whole genomic map of strain BaP3. The full length of the genome was 5.19 Mbp with the accession number JAPFGF000000000.

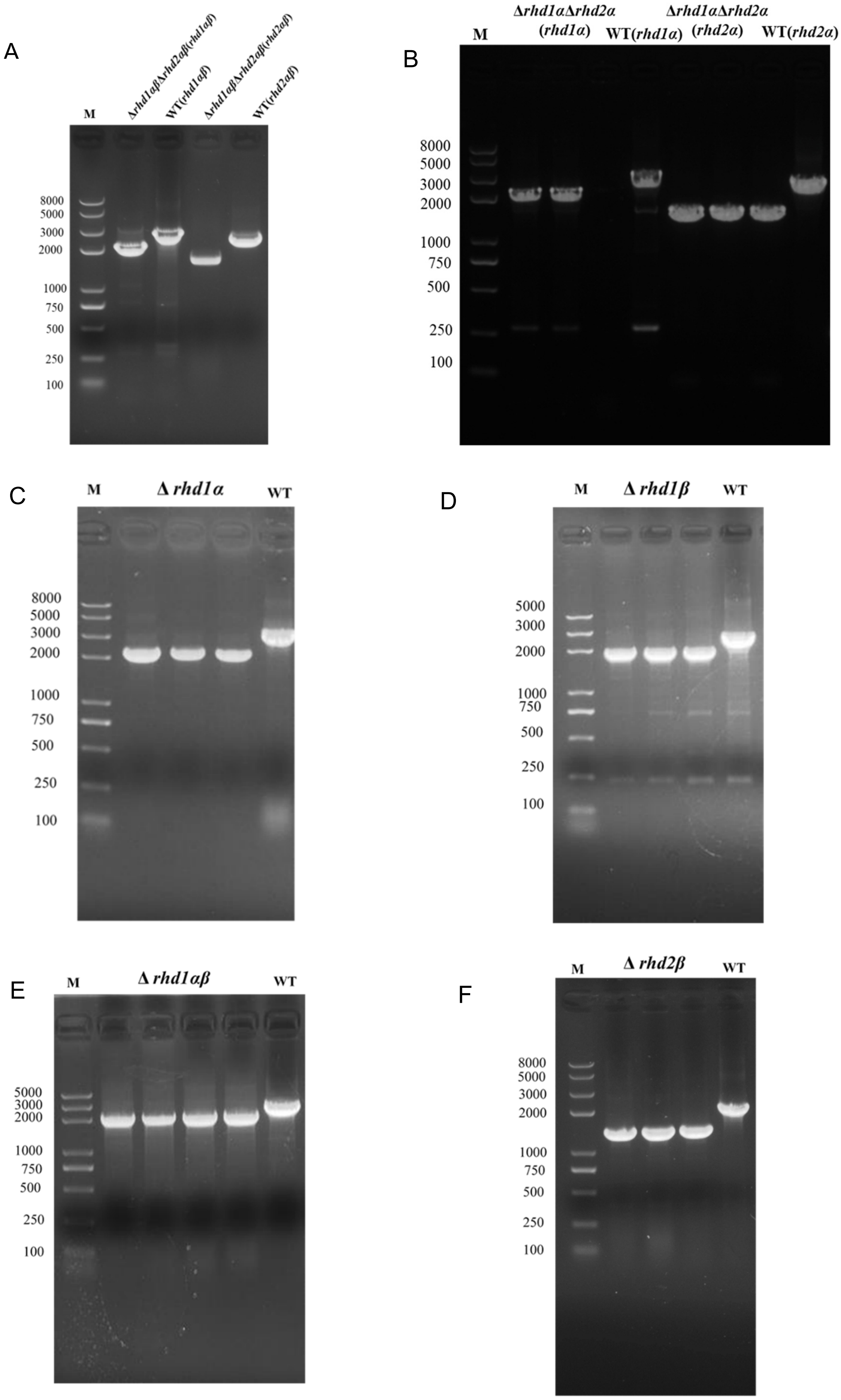
A
M
Δrhd1αβΔrhd2αβ(rhd1αβ)
WT(rhd1αβ)
Δrhd1αβΔrhd2αβ(rhd2αβ)
WT(rhd2αβ)
8000
5000
3000
2000
1000
750
500
250
100
B
M
Δrhd1αΔrhd2α (rhd1α)
WT(rhd1α)
Δrhd1αΔrhd2α (rhd2α)
WT(rhd2α)
8000
5000
3000
2000
1000
750
500
250
100
C
M
Δ rhd1α
WT
8000
5000
3000
2000
1000
750
500
250
100
D
M
Δ rhd1β
WT
5000
3000
2000
1000
750
500
250
100
E
M
Δ rhd1αβ
WT
5000
3000
2000
1000
750
500
250
100
F
M
Δ rhd2β
WT
8000
5000
3000
2000
1000
750
500
250
100

G

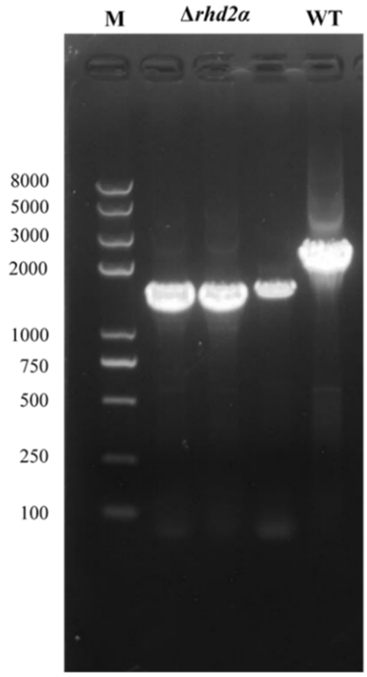

H

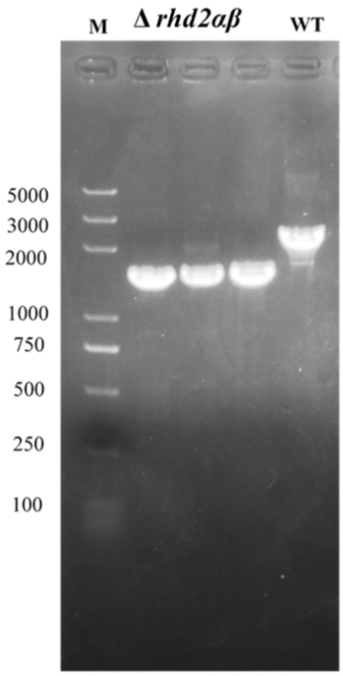

Figure S2. The identifications of knock-out mutant strains. The fragments of mutants were shorter than wild type BaP3. The strain Δ*rhd1αβ*Δ*rhd2αβ* (**A**), Δ*rhd1α*Δ*rhd2α* (**B**), Δ*rhd1α*(**C**), Δ*rhd1β* (**D**), Δ*rhd1αβ* (**E**), Δ*rhd2α*(**F**), Δ*rhd2β* (**G**), and Δ*rhd2αβ* (**H**) were obtained.

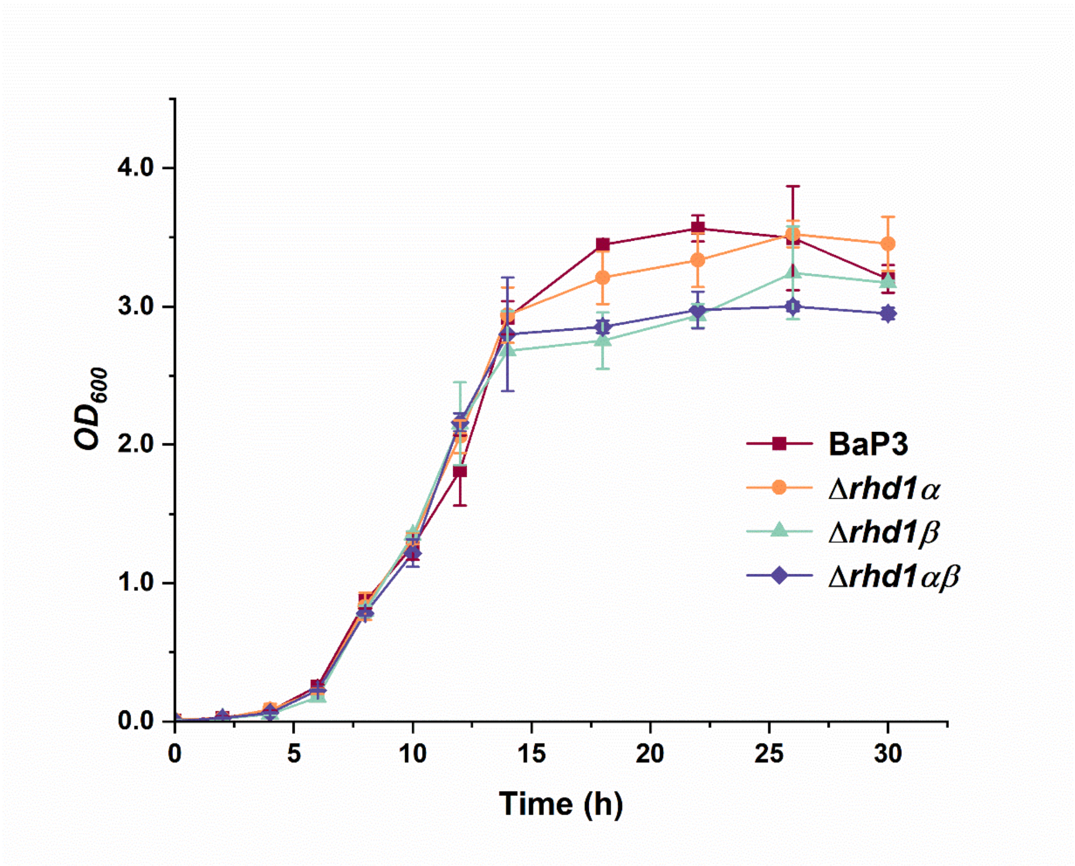

Figure S3. The growth curves in LB medium. The growth curves were obtained in 50 mL LB medium, samples were taken every 2 h and measured the absorbance at 600 nm. The gene deletion strains and wild type showed a similar growth rate in LB medium. The values proposed were the average ± standard deviation of a least three times of independent experiments.

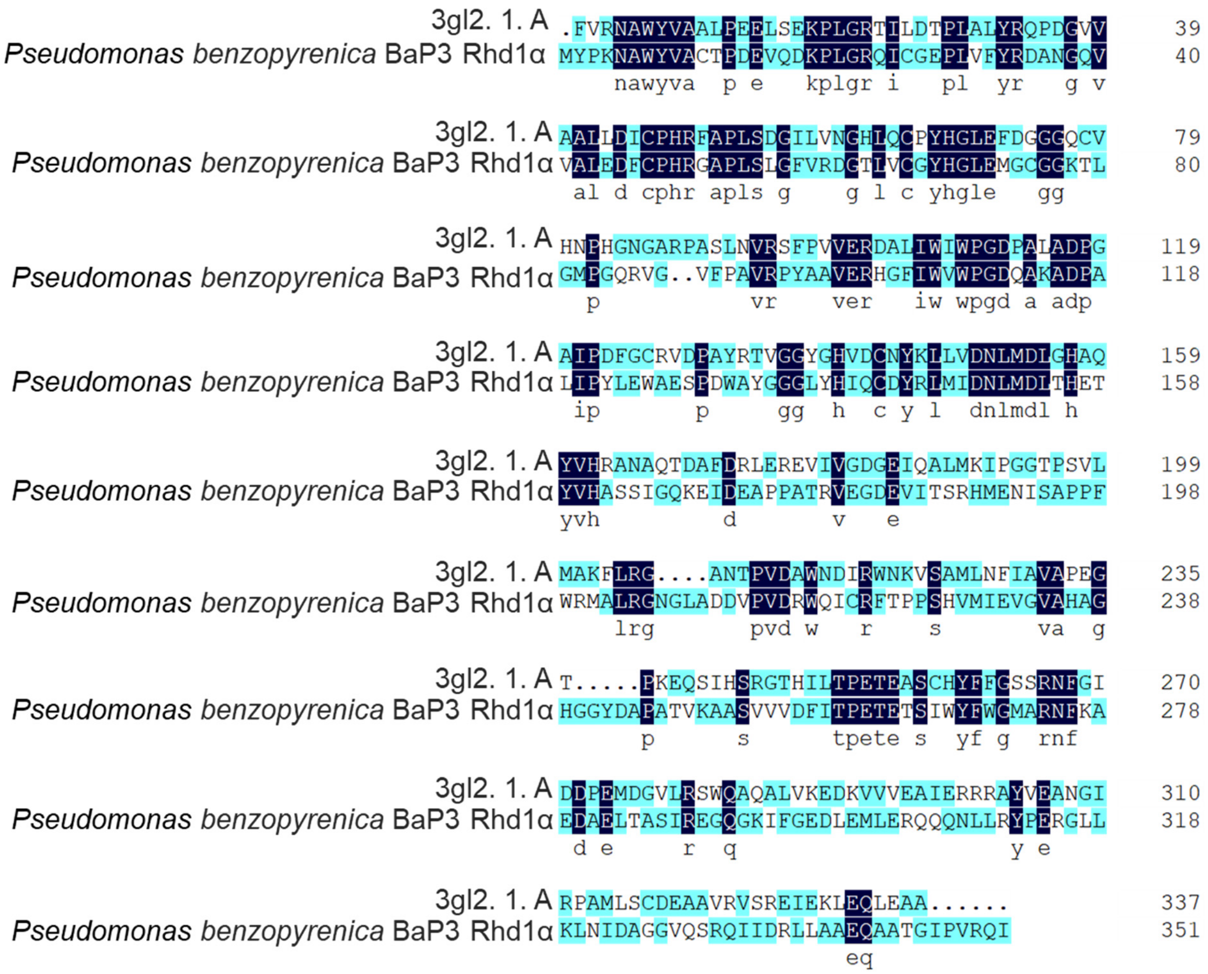

Figure S4. The multiple alignment of 3gl2. 1. A and Rhd1α, they shared 37.46% similarity. To form a hydrophobic substrate binding cavity, some residues was changed from hydrophilic amino acid to hydrophobic amino acid (Arg163 to Ala162, Glu177 to Ala176, Pro273 to Ala281, and Gly277 to Ala285) or become more hydrophobic (Ala166 to Ile165).

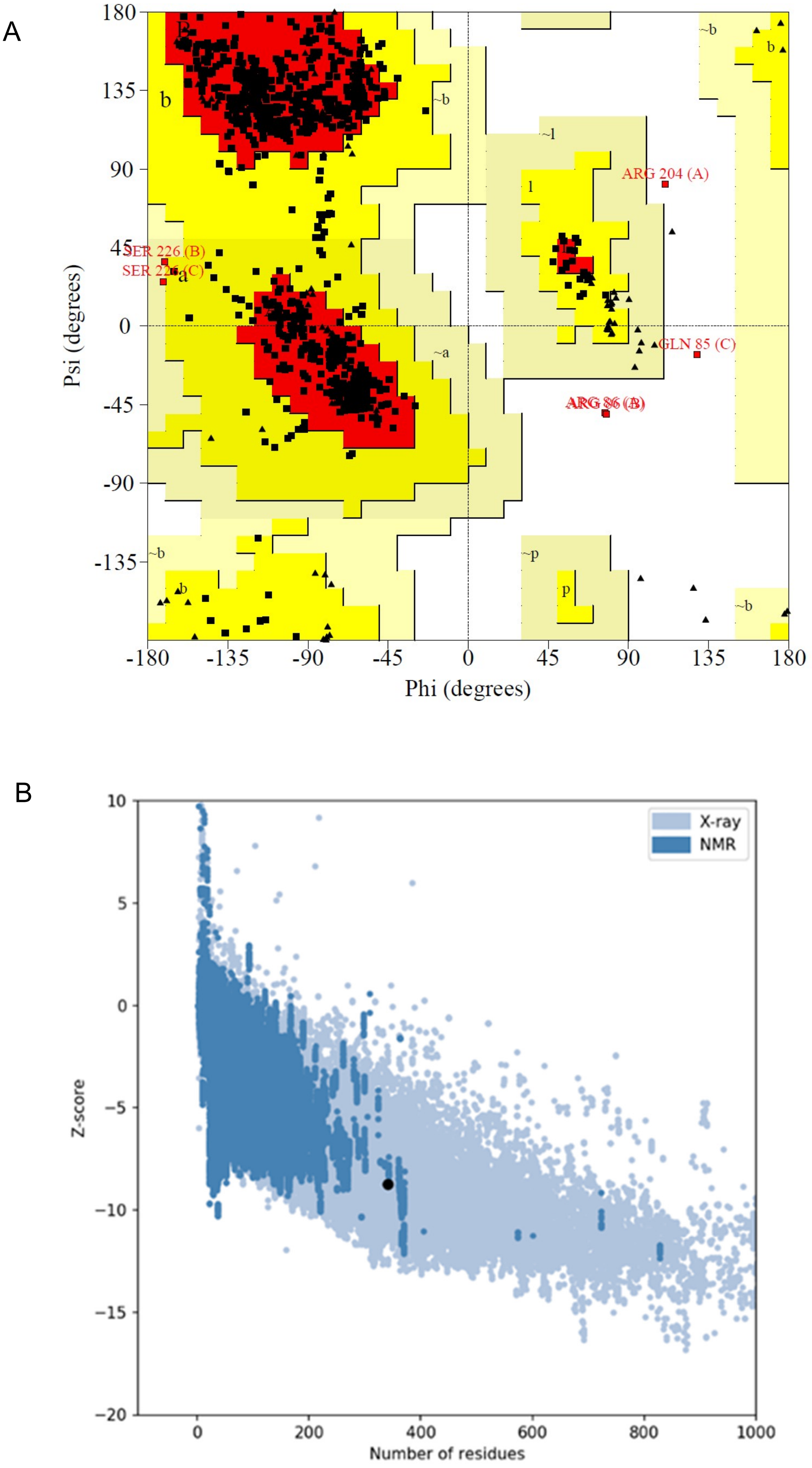

Figure S5. The Ramachandran plot of Rhd1α (A) and Z-Score analysis (B). According to the Ramachandran plot, 99.3% of residues were located in most favored or allowed regions. The Z-Score was -8.74. It identified that the model of Rhd1α was reasonable and stable.

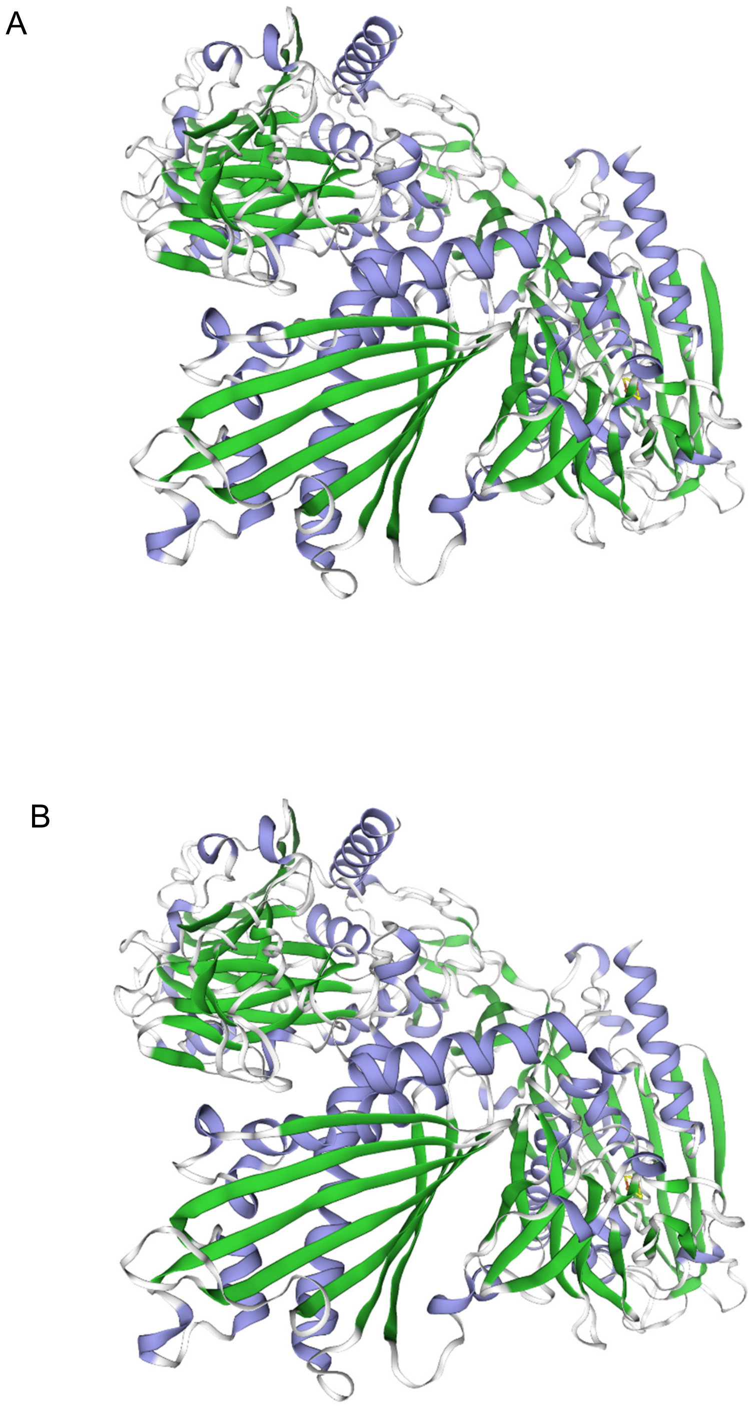
A
B

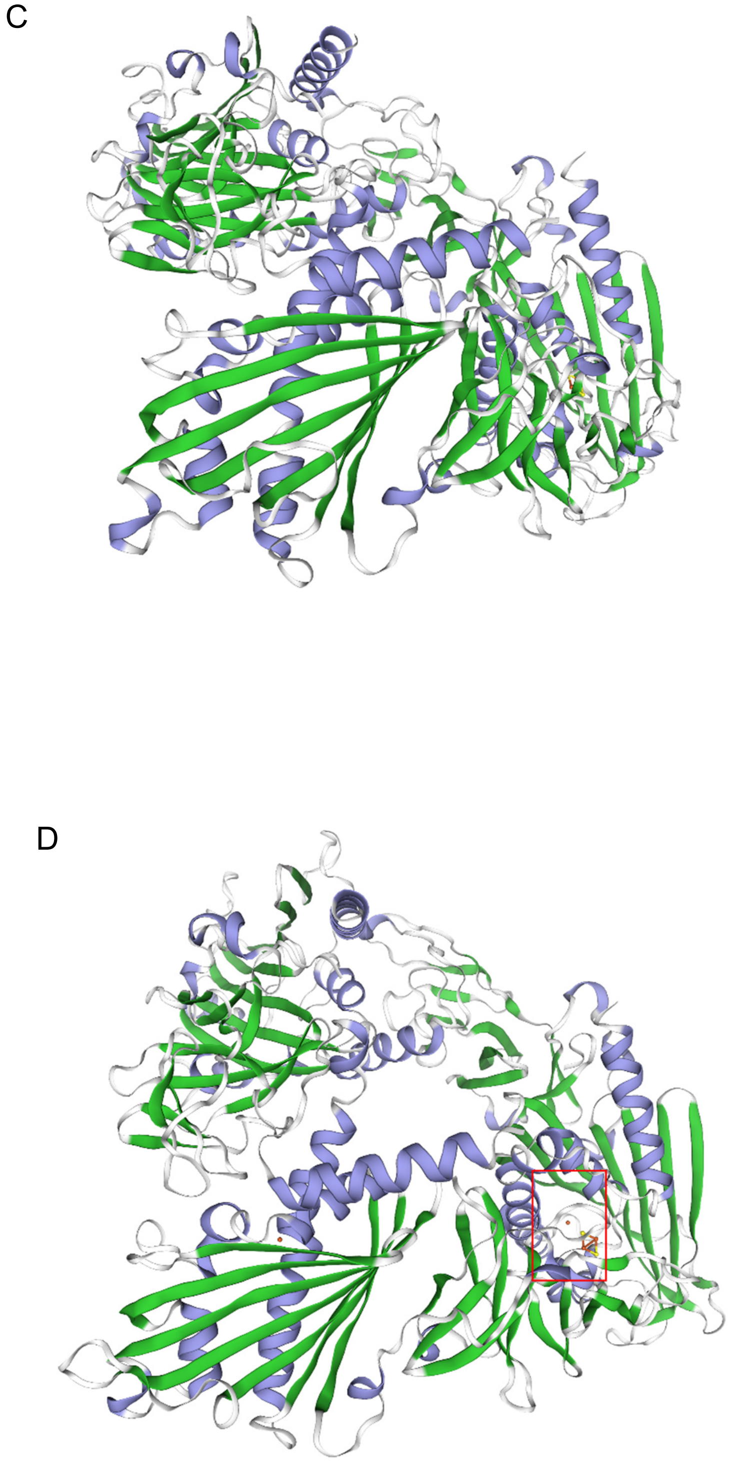

Figure S6. The structures of point-mutated strains (A–D). The active site could not be formed due to the point substitution of H156, H161, and D298 to Ala, while the substitution of Y159 to Ala could still construct the active site. The point mutations of H156, H161, and D298 to Ala caused the active site could not to be formed, while the mutation of Y159 to Ala could still construct the active site (yellow atoms in a red box at the bottom right-hand corner of the Figure S6D).

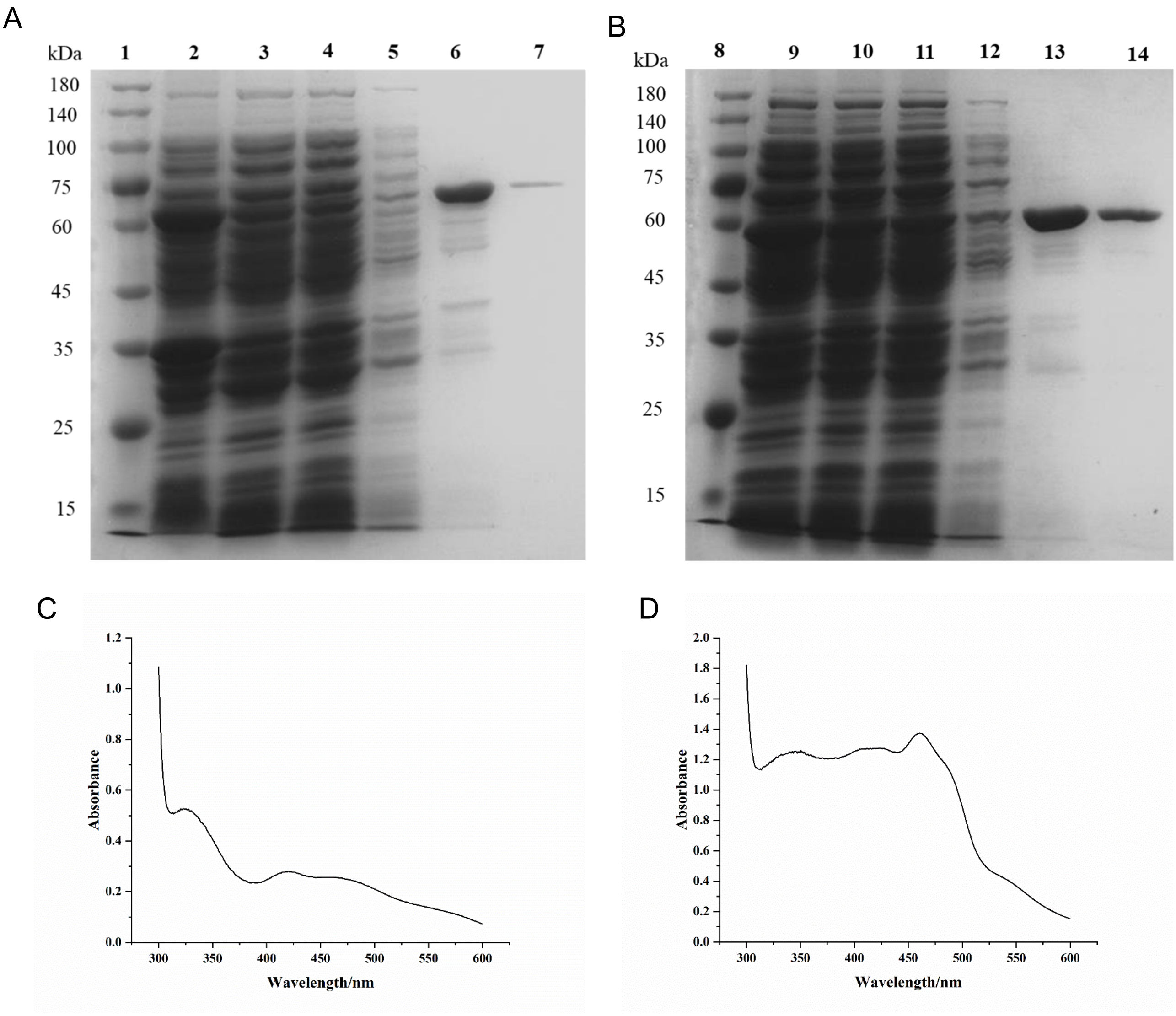

Figure S7. The SDS-PAGE of purified protein Rhd1α (**A**) and Rhd1β (**B**). The ultraviolet scanning of purified Rhd1α (**C**), and Rhd1β (**D**). The SDS-PAGE showed the purification process of Rhd1α and Rhd1β. Lane 1 and 8 were the protein marker (molecular weight was shown on the left). Lane 2 and 9 were the supernatant after ultrasonic. Lane 3,4 and 10,11 were the flow through liquid after passing the column. Lane 5 and 12 were to wash the impurity proteins by Tris/NaCl buffer. Lane 6,7 and 13,14 were the eluted target proteins liquid. The purified Rhd1α protein had absorption peaks at 323 nm, 420 nm, and 454nm. For Rhd1β, it had absorption peaks at 345 nm, 423 nm, 460 nm, 485 nm (shoulder), and 548 nm (shoulder).

Table S1. Bacterial strains used in this study.

Strains	Description[a]	Source
Escherichia coli S17-1 (λpir)	*thi*, *pro*, *hsd*R, *rec*A; chromosomal RP4-2; (Tc::Mu) (Km::Tn7) Tp^r, Sp^r	Simon, et al. [1]
E. coli DH5α	F^-, φ80(*lac*Z)ΔM15, Δ(*lac*ZYA-*argF*) U169, *deo*R, *rec*A1, *end*A1, *hsd*R17 (rK^-, mK^+), *pho*A, *sup*E44 λ^-, *thi*-1 *gyr*A96, *rel*A1	Laboratory
E. coli BL21 (DE3)	F^-, *omp*T, *hsd*S (r_B^- m_B^-), *gal*, *dcm* (DE3)	Laboratory
Pseudomonas benzopyrenica BaP3	Wild-type, Cm^r	Laboratory
P. donghuensis HYS	Wild-type, Cm^r	Laboratory
BL21-1	BL21/pGEX-rhd1α	This study
BL21-2	BL21/pGEX-rhd1β	This study
BaP3-1	Δ*rhd1α*Δ*rhd2α*	This study
BaP3-2	Δ*rhd1α*Δ*rhd2α*/pBBR2	This study
BaP3-3	Δ*rhd1α*Δ*rhd2α*/pBBR2-rhd1α	This study
BaP3-4	Δ*rhd1α*Δ*rhd2α*/pBBR2-rhd2α	This study
BaP3-5	Δ*rhd1αβ*Δ*rhd2αβ*	This study
BaP3-6	Δ*rhd1αβ*Δ*rhd2αβ*/pBBR2	This study
BaP3-7	Δ*rhd1αβ*Δ*rhd2αβ*/pBBR2-rhd1	This study
BaP3-8	Δ*rhd1αβ*Δ*rhd2αβ*/pBBR2-rhd2	This study
BaP3-9	Δ*rhd1α*	This study
BaP3-10	Δ*rhd1α*/pBBR2-rhd1α	This study
BaP3-11	Δ*rhd1α*/pBBR2	This study
BaP3-12	Δ*rhd1α*/pBBR2-H156A	This study
BaP3-13	Δ*rhd1α*/pBBR2-H161A	This study
BaP3-14	Δ*rhd1α*/pBBR2-D298A	This study
BaP3-15	Δ*rhd1α*/pBBR2-Y159A	This study
BaP3-16	Δ*rhd1β*	This study
BaP3-17	Δ*rhd1β*/pBBR2-rhd1β	This study
BaP3-18	Δ*rhd1αβ*	This study
BaP3-19	Δ*rhd1αβ*/pBBR2-rhd1αβ	This study
BaP3-20	Δ*rhd2α*	This study
BaP3-21	Δ*rhd2α*/pBBR2-rhd2α	This study
BaP3-22	Δ*rhd2β*	This study
BaP3-23	Δ*rhd2β*/pBBR2-rhd2β	This study
BaP3-24	Δ*rhd2αβ*	This study
BaP3-25	Δ*rhd2αβ*/pBBR2-rhd2αβ	This study
HYS-1	HYS/pBBR2-rhd1αβ	This study
HYS-2	HYS/pBBR2	This study

[a] Cm, chloramphenicol, Gm, gentamicin, Km, kanamycin;.

Table S2. Plasmids used in this study

Plasmids	Description[a]	Source
pEX18Gm	Gene replacement vector, Gm^r, *ori*T^+, *sac*B^+	Hoang, et al. [2]
pEX18-rhd1α	Gene replacement vector for *rhd1α*	This study
pEX18-rhd1β	Gene replacement vector for *rhd1β*	This study
pEX18-rhd1αβ	Gene replacement vector for *rhd1αβ*	This study
pEX18-rhd2α	Gene replacement vector for *rhd2α*	This study
pEX18-rhd2β	Gene replacement vector for *rhd2β*	This study
pEX18-rhd2αβ	Gene replacement vector for *rhd2αβ*	This study
pBBR1-MCS2	Mobilizable broad-host-range cloning vector, Km^r	Kovach et al. [3]
pBBR2-rhd1α	Cloning vector for *rhd1α*	This study
pBBR2-rhd1β	Cloning vector for *rhd1β*	This study
pBBR2-rhd1αβ	Cloning vector for *rhd1αβ*	This study
pBBR2-rhd2α	Cloning vector for *rhd2α*	This study
pBBR2-rhd2β	Cloning vector for *rhd2β*	This study
pBBR2-rhd2αβ	Cloning vector for *rhd2αβ*	This study
pBBR2-H156A	Point mutation vector of H156	This study
pBBR2-H161A	Point mutation vector of H161	This study
pBBR2-D298A	Point mutation vector of D298	This study
pBBR2-Y159A	Point mutation vector of Y159	This study
pGEX-4T-1	Protein expression vector, Am^r	Gifted by Prof. Xiangdong Gao
pGEX-rhd1α	Heterologous expression of *rhd1α*	This study
pGEX-rhd1β	Heterologous expression of *rhd1β*	This study

[a] Gm, gentamicin, Km, kanamycin, Am, ampicillin.

Table S3. Primers used in this research. The engineered restriction sites were underlined.

Primer	Sequence (5′-3′)	Purpose
rhd1α-up-F	CGGAATTCCGGCCGTCGATCATGTTGAGCA	Construction of pEX18-rhd1α pEX18-rhd1β pEX18-rhd1αβ and identification
rhd1α-overlap-R	GATATTGAGCTTGAGCAGCCCGCGGACGAAGCCCAAGGA	
rhd1α-overlap-F	TCCTTGGGCTTCGTCCGCGGGCTGCTCAAGCTCAATATC	
rhd1α-down-R	CCCAAGCTTGGGTGAGCCAGGGGATGAAGG	
rhd1α-WF	ACCAGCGGGATGGCCCCCAG	
rhd1α-WR	GCGGCGTGCTCGGCTTCGGAAAGAAA	
rhd1β-up-F	CGGAATTCCGATGGCGCTACGTGGCAATGG	
rhd1β-overlap-R	GGTCAGGCAGGTGCCGCAGACGGTGCAGAGCGAATAGTGG	
rhd1β-overlap-F	CCACTATTCGCTCTGCACCGTCTGCGGCACCTGCCTGACC	
rhd1β-down-R	CCCAAGCTTGGGGCGCCGAGGCGATCTGCTGTT	
rhd1β-WF	GCAAGACCCTGGGCATGCCA	
rhd1β-WR	GCAAGCCGGGACAGCGGGTA	
rhd1αβ-up-F	CGGAATTCCGAAGGCCATTCGGGACCGGAT	
rhd1αβ-WF	AGCATGCCGAGCCCCGAGAG	
rhd2α-up-F	CCCAAGCTTGGGAGCCCGCTCATCGACTCGGA	Construction of pEX18-rhd2α pEX18-rhd2β pEX18-rhd2αβ and identification
rhd2α-overlap-R	GCGATGGGTGATGGCGACAAGCGCCCGGATGTTCTCGTCGTC	
rhd2α-overlap-F	GACGACGAGAACATCCGGGCGCTTGTCGCCATCACCCATCGC	
rhd2α-down-R	CGGAATTCCGGGGGGTACGGGCACTGTGGAC	
rhd2α-WF	ACGAAGAGGTCTTTCCGGTC	
rhd2α-WR	CCTCACAGATGGCGAAAACCTT	
rhd2β-up-F	GCTCTAGAGCGGTCATCGCCAATCATGGCGAATC	
rhd2β-overlap-R	GCCACAGGAGGAAGGCACGATGGTGTAGCACCGCGAGAGACT	
rhd2β-overlap-F	AGTCTCTCGCGGTGCTACACCATCGTGCCTTCCTCCTGTGGC	
rhd2β-down-R	CGGAATTCCGCGCCAGCGTGCAGGATGAAATCG	
rhd2β-WF	GGGAAGACCAAGGCCACCTCTGTGC	
rhd2β-WR	GCCCAGGGCACGTCGTCGTT	
rhd2αβ-up-F	GCTCTAGAGCCCATGGCTCATGCCAGCGTA	
rhd2αβ-WF	GCGACGCCTATTACCTGGTGACACCCCAT	
rhd1α-F	GGGGTACCCCATCGATTATTGGATCCAATAGACAA	Construction of pBBR2-rhd1α pBBR2-rhd1β pBBR2-rhd1
rhd1α-R	GCTCTAGAGCGAGCATGACTGTCTCCTAGG	
rhd1β-F	CCCTCGAGGGGCCTAGGAGACAGTCATGCT	
rhd1β-R	GCTCTAGAGCGGGCTCGGGTCATCAGAGGT	
rhd2α-F	GGGGTACCCCCACTTCGATACTGCGGCC	Construction of pBBR2-rhd2α pBBR2-rhd2β pBBR2-rhd2
rhd2α-R	GCTCTAGAGCTGAGGGTCGTCATGGTATTC	
rhd2β-F	GGGGTACCCCGTGAGGAATACCATGACGACCC	
rhd2β-R	GCTCTAGAGCTACCGATGCAGGCGCGTTG	
His156-F	ATGGATCTCACCGCCGAGACCTACGTCCAT	Construction of H156A
His156-R	ATGGACGTAGGTCTCGGCGGTGAGATCCAT	
His161-F	CACGAGACCTACGTCGCTGCCTCCAGCATC	Construction of H161A
His161-R	GATGCTGGAGGCAGCGACGTAGGTCTCGTG	
Asp298-F	AGGGCAAGATCTTCGGCGAAGCCCTGGAGA	Construction of D298A
Asp298-R	TCTCCAGGGCTTCGCCGAAGATCTTGCCCT	
Try159-F	CACGAGACCGCCGTCCATGCCTCCAGCATC	Construction of Y159A
Try159-R	GATGCTGGAGGCATGGACGGCGGTCTCGTG	
Rhd1α-Pr-F	CCGGAATTCCGGATGTACCCGAAGAATGCCTGGTA	Construction of pGEX-rhd1α
Rhd1α-Pr-R	CCCTCGAGGGCTAGGCGATCTGCCGGACC	
Rhd1β-Pr-F	CGGAATTCCCAGTCATGCTCGATCTGATCGTCACC	Construction of pGEX-rhd1β
Rhd1β-Pr-R	CCCTCGAGGGTCAGAGGTCCAGTACCAGGCGC	

References

1. Simon, R.; Priefer, U.; Pühler, A. A Broad Host Range Mobilization System for In Vivo Genetic Engineering: Transposon Mutagenesis in Gram Negative Bacteria. *Bio/Technology* **1983**, *1*, 784–791. https://doi.org/10.1038/nbt1183-784.
2. Hoang, T.T.; Karkhoff-Schweizer, R.R.; Kutchma, A.J.; Schweizer, H.P. A broad-host-range Flp-FRT recombination system for site-specific excision of chromosomally-located DNA sequences: application for isolation of unmarked Pseudomonas aeruginosa mutants. *Gene* **1998**, *212*, 77–86. https://doi.org/10.1016/s0378-1119(98)00130-9.
3. Kovach, M.E.; Elzer, P.H.; Hill, D.S.; Robertson, G.T.; Farris, M.A.; Roop, R.M.; Peterson, K.M. Four new derivatives of the broad-host-range cloning vector pBBR1MCS, carrying different antibiotic-resistance cassettes. *Gene* **1995**, *166*, 175–176. https://doi.org/10.1016/0378-1119(95)00584-1.
